# The impact of low body mass index on clinical outcomes after coronary artery bypass graft surgery

**DOI:** 10.1016/j.xjon.2025.08.003

**Published:** 2025-08-25

**Authors:** Yuki Kuroda, Hiroki Shiomi, Takeshi Morimoto, Takeshi Shimamoto, Takehiko Matsuo, Koh Ono, Takeshi Kimura, Kenji Minatoya

**Affiliations:** a Department of Cardiovascular Surgery, Kyoto University Graduate School of Medicine, Kyoto, Japan; b Department of Cardiovascular Medicine, Kyoto University Graduate School of Medicine, Kyoto, Japan; c Department of Data Science, Hyogo Medical University, Nishinomiya, Japan; d Department of Cardiology, Hirakata Kohsai Hospital, Hirakata, Japan

**Keywords:** coronary artery bypass grafting, body mass index, underweight, sex differences

## Abstract

**Objective:**

To clarify the effects of being underweight on clinical outcomes after coronary artery bypass grafting (CABG) and possible associated sex differences.

**Methods:**

The study population included 5914 patients who underwent their first isolated CABG; patients with acute myocardial infarction were excluded. Clinical outcomes within and beyond 30 days after CABG were compared across groups on the basis of preoperative body mass index (BMI): underweight (BMI <18.5; n = 318), normal (18.5 ≤ BMI < 25; n = 3835), overweight (25 ≤ BMI < 30; n = 1580), and obese (BMI ≥30; n = 181).

**Results:**

The cumulative 30-day incidence of all-cause death was 3.2%, 1.2%, 0.4%, and 1.1% in the underweight, normal, overweight, and obese groups, respectively (log-rank *P* < .001). This trend was more prominent in men than in women (4.0%, 1.3%, 0.4%, and 1.6%, log-rank *P* < .001; 1.7%, 0.9%, 1.1%, and 0.0%, log-rank *P* = .74). The cumulative 5-year incidence of all-cause death >30 days after CABG was significantly greater in the underweight group (27.1%, 16.6%, 10.1%, and 6.5%; log-rank *P* < .001). The greater risk of being underweight and the lower risk of being overweight or obese relative to normal were significant for all-cause death (adjusted hazard ratio, 1.22 [95% confidence interval, 1.00-1.49]; 0.77 [0.68-0.89]; and 0.63 [0.42-0.95], respectively). Furthermore, the excess mortality risk of being underweight relative to normal was significant in men (1.32 [1.03-1.68]) but not in women (1.14 [0.80-1.63]) (interaction *P* = .01).

**Conclusions:**

Being underweight was associated with increased short- and long-term mortality after CABG, especially in men, whereas being overweight or obese was associated with decreased long-term mortality after CABG.

## Graphical Abstract

**Figure:**
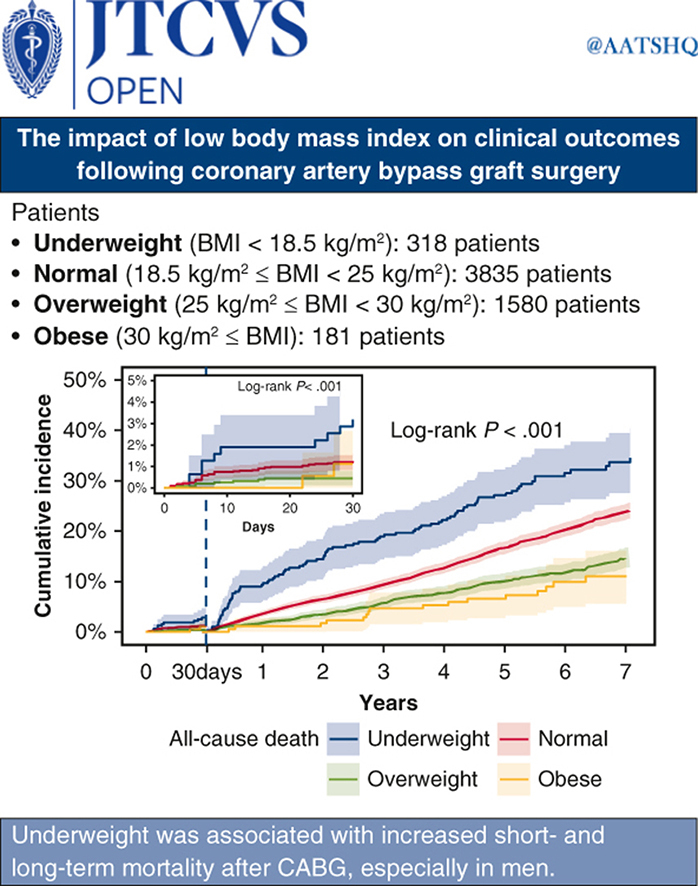

**Figure:**
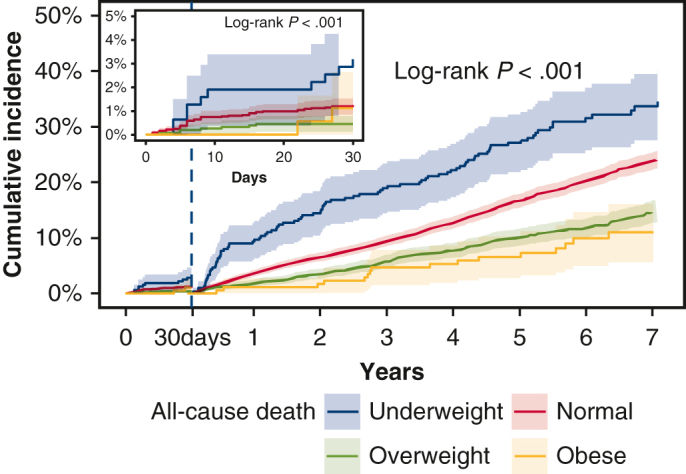
Having underweight was associated with increased mortality after CABG, especially in men.

**Central Message**

Having underweight was associated with increased short- and long-term mortality risk after CABG, driven primarily by noncardiovascular death, particularly in men.

**Perspective**

The impact of underweight on long-term clinical outcomes after CABG has been poorly documented and data on associated sex differences were lacking. This study showed that having underweight was associated with increased mortality after CABG, especially in men. Preoperative nutritional optimization and enhanced post-CABG surveillance could improve outcomes in patients with underweight.

Obesity is a global health issue and a known risk factor for cardiovascular diseases, including atherosclerosis, diabetes, hypertension, heart failure, and atrial fibrillation. Nevertheless, it has been suggested that patients with obesity who develop cardiovascular diseases have better prognoses than patients without obesity.^1^^,^^2^ This “obesity paradox” has been identified partially in patients undergoing coronary artery bypass grafting (CABG): patients with overweight and with mild obesity are reported to have improved early and intermediate survival after CABG.^3^

In contrast, being underweight is reported to be a predictor of early mortality among patients undergoing isolated CABG.^4^ However, the impact of being underweight on long-term clinical outcomes in patients who undergo CABG has been poorly documented. Furthermore, the differences in its impact between men and women have not yet been adequately evaluated. Therefore, we sought to clarify the effect of being underweight on clinical outcomes in patients undergoing CABG and the associated sex differences using a large-scale, multicenter registry in Japan.

## Methods

### Study Population

The CREDO-Kyoto (Coronary Revascularization Demonstrating Outcome Study in Kyoto) percutaneous coronary intervention (PCI)/CABG registries were a series of physician-initiated, noncompany-sponsored, multicenter registries enrolling consecutive patients who underwent their first coronary revascularization during 3 separate time periods in the past 2 decades in Japan.^5^, ^6^, ^7^ The relevant ethics committees in all participating centers (Appendix E1) approved the study protocol. Because of the retrospective nature of the study, the requirement for written informed consent was waived, although we excluded patients who refused participation in the study when contacted for follow-up. This strategy was concordant with the guidelines of the Japanese Ministry of Health, Labor and Welfare.

**Figure 1:**
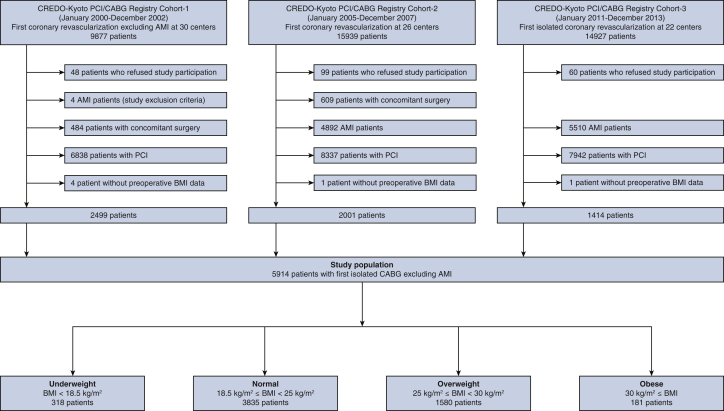
Study flowchart. Concomitant surgery indicates combined noncoronary surgery. *PCI*, Percutaneous coronary intervention; *CABG*, coronary artery bypass grafting; *AMI,* acute myocardial infarction; *BMI*, body mass index.

The CREDO-Kyoto PCI/CABG Registry cohort 1 enrolled 9877 patients across 30 centers between January 2000 and December 2002 (bare-metal stent era), cohort 2 enrolled 15,939 patients across 26 centers between January 2005 and December 2007 (first-generation drug-eluting stent era), and cohort 3 enrolled 14,927 patients across 22 centers between January 2011 and December 2013 (new-generation drug-eluting stent era). Patients who had acute myocardial infarction within 1 week before the index procedure were not enrolled in cohort 1 but were enrolled in cohorts 2 and 3. From the 40,743 patients enrolled in the CREDO-Kyoto PCI/CABG Registry cohorts 1, 2, and 3, we excluded 207 patients who declined study participation, 1093 patients with concomitant surgery, 10,406 patients with acute myocardial infarction, 23,117 patients who underwent PCI, and 6 patients who lacked data on preoperative body mass index (BMI). Thus, the current study population consisted of 5914 patients who underwent their first isolated CABG (Figure 1). Among them, patients were categorized on the basis of preoperative BMI data; underweight: BMI <18.5 kg/m^2^, normal: 18.5 kg/m^2^ ≤ BMI <25 kg/m^2^, overweight: 25 kg/m^2^ ≤ BMI <30 kg/m^2^, and obese: BMI ≥30 kg/m^2^.^8^

### Data Collection for Baseline Characteristics and Follow-up Clinical Events

Clinical, angiographic, and procedural data were collected from hospital charts or hospital databases according to prespecified definitions by experienced clinical research coordinators from the Research Institute for Production Development (Kyoto, Japan) (Appendix E2). Definitions for clinical characteristics were identical across the 3 cohorts (Appendix E3).

Follow-up data were collected from hospital charts or obtained through contact with patients, their relatives, or the referring physicians. The primary outcome measure in this study was all-cause death after CABG. The secondary outcome measures included cardiovascular death, noncardiovascular death, myocardial infarction, heart failure, stroke, and repeat coronary revascularization. All definitions for clinical outcome measures were identical across the three cohorts (Appendix E3).

### Statistical Analyses

Categorical variables are presented as number (percentage) and compared using the χ^2^ test. Continuous variables are presented as mean (standard deviation) or median (interquartile range [IQR]) and compared using analysis of variance or the Kruskal-Wallis test. Time zero for clinical follow-up was defined as the day of CABG. Cumulative incidences of the clinical outcomes were estimated using the Kaplan-Meier method, and differences were assessed using the log-rank test. We conducted a landmark analysis at 30 days after CABG, given that the occurrence of clinical events might differ between the acute postoperative period and long-term follow-up. The effects of being underweight, overweight, or obese relative to normal for the outcome measures beyond 30 days after CABG were estimated by the Cox proportional hazard models and expressed as hazard ratios (HRs) and their 95% confidence intervals (CIs). The adjusted effects for outcome measures were estimated using the multivariable Cox proportional hazard models and adjusting for 14 clinically relevant factors, consistent with our previous studies (Table 1).^9^ We performed a stratified analysis by sex, and formal interactions between sex and the effect of BMI categories on clinical outcomes were assessed using the Cox proportional hazard models. Regarding missing data, we performed a complete-case analysis.

**Table 1:** Baseline characteristics and management

Characteristic	Underweight n = 318	Normal n = 3835	Overweight n = 1580	Obese n = 181	*P* value
Clinical characteristics					
Body mass index, kg/m^2^	17.5 (0.9)	22.3 (1.7)	26.7 (1.3)	32.3 (2.5)	<.001
Age, y	70.8 (9.4)	68.8 (9.0)	66.5 (9.4)	61.8 (12.3)	<.001
Age ≥75 y∗	125 (39%)	1089 (28%)	330 (21%)	32 (18%)	<.001
Men∗	200 (63%)	2850 (74%)	1220 (77%)	128 (71%)	<.001
Unstable angina	21 (6.6%)	218 (5.7%)	65 (4.1%)	3 (1.7%)	.01
Hypertension∗	219 (69%)	2940 (77%)	1305 (83%)	159 (88%)	<.001
Diabetes∗	141 (44%)	1872 (49%)	778 (49%)	93 (51%)	.37
On insulin therapy	56 (18%)	633 (17%)	207 (13%)	31 (17%)	.01
Current smoker	77 (24%)	843 (22%)	376 (24%)	43 (24%)	.47
Heart failure∗	66 (21%)	521 (14%)	206 (13%)	18 (9.9%)	.001
LVEF (%)	55.0 (15.4)	58.9 (14.5)	61.0 (13.0)	60.5 (13.2)	<.001
≤40%	58 (18%)	452 (12%)	120 (7.6%)	17 (9.4%)	<.001
Severe MR	16 (5.6%)	121 (3.4%)	36 (2.4%)	3 (1.7%)	.02
Previous myocardial infarction∗	104 (33%)	1070 (28%)	388 (25%)	44 (24%)	.01
Previous stroke∗	67 (21%)	689 (18%)	269 (17%)	26 (14%)	.20
Peripheral vascular disease∗	67 (21%)	659 (17%)	171 (11%)	26 (14%)	<.001
Atrial fibrillation	38 (12%)	396 (10%)	146 (9.2%)	21 (12%)	.38
eGFR, mL/min/1.73 m2	47.5 (25.2)	58.7 (26.0)	66.7 (27.1)	76.6 (38.1)	<.001
eGFR <30, without hemodialysis∗	27 (8.5%)	249 (6.5%)	76 (4.8%)	9 (5.0%)	.03
Hemodialysis∗	47 (15%)	261 (6.8%)	53 (3.4%)	4 (2.2%)	<.001
Anemia (hemoglobin <11.0 g/dL)∗	115 (36%)	782 (20%)	168 (11%)	18 (9.9%)	<.001
Chronic obstructive pulmonary disease∗	16 (5.0%)	133 (3.5%)	32 (2.0%)	3 (1.7%)	.005
Liver cirrhosis∗	14 (4.4%)	131 (3.4%)	34 (2.2%)	2 (1.1%)	.02
Malignancy∗	38 (12%)	358 (9.3%)	127 (8.0%)	13 (7.2%)	.10
Procedural characteristics					
Extent of coronary artery disease					.48
One-vessel disease	5 (1.6%)	79 (2.1%)	26 (1.6%)	4 (2.2%)	
Two-vessel disease	46 (14%)	479 (12%)	212 (13%)	24 (13%)	
Three-vessel disease	166 (52%)	2041 (53%)	883 (56%)	101 (56%)	
Left main coronary artery disease	101 (32%)	1236 (32%)	459 (29%)	52 (29%)	
Cardiopulmonary bypass use	152 (48%)	1719 (45%)	712 (45%)	88 (49%)	.59
Internal thoracic artery use	295 (93%)	3681 (96%)	1532 (97%)	172 (95%)	.005
Medications at discharge					
Aspirin	282 (89%)	3515 (92%)	1457 (92%)	176 (97%)	.01
Thienopyridine	36 (11%)	529 (14%)	202 (13%)	18 (9.9%)	.26
Cilostazol	21 (6.6%)	343 (9.0%)	111 (7.0%)	7 (3.9%)	.01
Statins	68 (21%)	1233 (32%)	617 (39%)	100 (55%)	<.001
ACE-I/ARB	76 (24%)	971 (25%)	447 (28%)	65 (36%)	.002
Beta-blockers	64 (20%)	950 (25%)	450 (29%)	57 (31%)	<.001
Nitrates	119 (38%)	1331 (35%)	526 (33%)	57 (31%)	.39
Calcium channel blockers	138 (44%)	1998 (52%)	829 (53%)	95 (52%)	.02
Oral anticoagulants	115 (36%)	1455 (38%)	563 (36%)	68 (38%)	.45
Warfarin	115 (36%)	1447 (38%)	560 (36%)	68 (38%)	.46
DOAC	0 (0%)	8 (0.2%)	3 (0.2%)	0 (0%)	.79

Continuous variables were expressed as mean (standard deviation). Categorical variables were expressed as number (percentage). Values were missing for diabetes mellitus in 2 patients, for insulin therapy in 15 patients, for current smoker in 56 patients, for heart failure in 12 patients, for LVEF in 442 patients, for severe MR in 435 patients, for previous myocardial infarction in 7 patients, for prior stroke in 1 patient, for peripheral vascular disease in 3 patients, for eGFR in 39 patients, for chronic obstructive pulmonary disease in 1 patient, for liver cirrhosis in 4 patients, for malignancy in 1 patient, for cardiopulmonary bypass use in 4 patients, for aspirin in 12 patients, for thienopyridine in 12 patients, for cilostazol in 12 patients, for statins in 12 patients, for ACE-I/ARB in 12 patients, for beta-blockers in 12 patients, for nitrates in 12 patients, for calcium channel blockers in 12 patients, and for warfarin in 12 patients. *LVEF*, Left ventricular ejection fraction; *MR*, mitral regurgitation; *eGFR*, estimated glomerular filtration rate; *ACE-I*, angiotensin-converting enzyme inhibitors; *ARB*, angiotensin II receptor blockers; *DOAC*, direct oral anticoagulant.

∗ Risk-adjusting variables selected for the Cox proportional hazard models.

All statistical analyses were conducted using R statistical software, version 4.4.1 (R Foundation for Statistical Computing, Vienna, Austria) and the following packages: gtsummary for baseline and regression table generation, survival for survival analysis, and ggplot2 for figures. All reported *P* values were 2-tailed.

## Results

### Baseline Characteristics

The 5914 patients who underwent CABG were categorized by BMI as follows: 318 (5.4%) in the underweight group (BMI <18.5 kg/m^2^), 3835 (65%) in the normal-BMI group (18.5 kg/m^2^ ≤ BMI < 25 kg/m), 1580 (27%) in the overweight group (25 kg/m^2^ ≤ BMI < 30 kg/m^2^), and 181 (3.1%) in the obese group (BMI ≥30 kg/m^2^) (Figure 1). The underweight group was characterized by having older patients; more women; more patients with heart failure, end-stage renal disease, and malignancy; and fewer patients with hypertension and diabetes (Table 1). Procedural characteristics were similar across the 4 groups. Regarding medications at discharge, the prescription rates of statins were lower in the underweight group than in the other groups.

### Clinical Outcomes Within 30 Days After CABG

The cumulative 30-day incidence of all-cause death was greater in the underweight group than in the other groups (underweight: 3.2%, normal: 1.2%, overweight: 0.4%, and obese: 1.1%; log-rank *P* < .001) (Table 2). The cumulative 30-day incidence of myocardial infarction was numerically greater in the underweight group than in the other groups (underweight: 4.4%, normal: 2.6%, overweight: 2.7%, and obese: 1.1%; log-rank *P* = .15). There were no differences across the four groups in the incidences of heart failure, stroke, or repeat coronary revascularization.

**Table 2:** Clinical outcomes within 30 days after CABG

Outcome	Group	n	Number of patients with event	Cumulative 30-d incidence	Log-rank *P* value
All-cause death					<.001
	Normal	3835	47	1.2%	
	Underweight	318	10	3.2%	
	Overweight	1580	7	0.4%	
	Obese	181	2	1.1%	
Cardiovascular death					<.001
	Normal	3835	47	1.2%	
	Underweight	318	10	3.2%	
	Overweight	1580	7	0.4%	
	Obese	181	2	1.1%	
Noncardiovascular death					NA
	Normal	3835	0	0.0%	
	Underweight	318	0	0.0%	
	Overweight	1580	0	0.0%	
	Obese	181	0	0.0%	
Myocardial infarction					.15
	Normal	3835	101	2.6%	
	Underweight	318	14	4.4%	
	Overweight	1580	43	2.7%	
	Obese	181	2	1.1%	
Heart failure					.34
	Normal	3835	13	0.3%	
	Underweight	318	2	0.6%	
	Overweight	1580	2	0.1%	
	Obese	181	1	0.6%	
Stroke					.90
	Normal	3835	63	1.7%	
	Underweight	318	6	1.9%	
	Overweight	1580	28	1.8%	
	Obese	181	2	1.1%	
Repeat coronary revascularization					.87
	Normal	3835	121	3.2%	
	Underweight	318	11	3.6%	
	Overweight	1580	49	3.1%	
	Obese	181	4	2.2%	

*CABG*, Coronary artery bypass grafting.

### Clinical Outcomes Beyond 30 Days After CABG

The median clinical follow-up after surgery was 5.8 (IQR, 4.5-8.8) years (underweight: 5.2 [IQR, 2.4-7.0] years, normal: 5.8 [4.4-8.2] years, overweight: 6.1 [4.8-10.4] years, and obese: 6.1 [4.8-8.8] years; *P* < .001).

**Figure 2:**
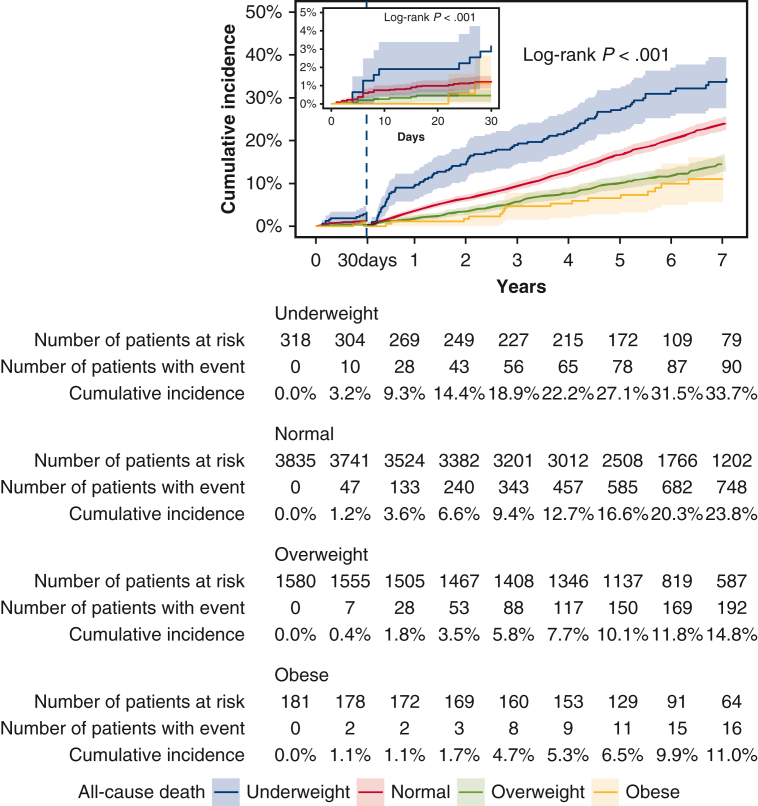
Kaplan-Meier event curves for all-cause death within and beyond 30 days after coronary artery bypass grafting. 95% confidence intervals are shown via *shading*.

The cumulative 5-year incidence of all-cause death beyond 30 days after CABG was significantly greater in the underweight group and lower in the overweight and obese groups compared with the normal group (underweight: 27.1%, normal: 16.6%, overweight: 10.1%, and obese: 6.5%; log-rank *P* < .001) (Table 3 and Figure 2). Even after adjusting for confounders, the greater risk of the underweight group relative to the normal group remained significant for all-cause death (adjusted HR, 1.22; 95% CI, 1.00-1.49; *P* = .048). The lower risk of the overweight and obese groups also remained significant for all-cause death (adjusted HR, 0.77; 95% CI, 0.68-0.89; *P* < .001, and adjusted HR, 0.63; 95% CI, 0.42-0.95; *P* = .03) (Table 3).

**Table 3:** Clinical outcomes beyond 30 days after CABG

Outcome	Group	N	Number of patients with event	Cumulative 5-year incidence	Log-rank *P* value	Crude HR (95% CI)	*P* value	Adjusted HR (95% CI)	*P* value
All-cause death					<.001				
	Normal	3737	997	16.6%		−		−	
	Underweight	303	113	27.1%		1.66 (1.37-2.02)	<.001	1.22 (1.00-1.49)	.048
	Overweight	1553	286	10.1%		0.63 (0.55-0.72)	<.001	0.77 (0.68-0.89)	<.001
	Obese	178	24	6.5%		0.47 (0.32-0.71)	<.001	0.63 (0.42-0.95)	.03
Cardiovascular death					<.001				
	Normal	3737	525	9.3%		−		−	
	Underweight	303	53	14.2%		1.47 (1.11-1.95)	.01	1.02 (0.77-1.37)	.87
	Overweight	1553	170	6.4%		0.71 (0.60-0.85)	<.001	0.91 (0.76-1.08)	.28
	Obese	178	15	4.7%		0.56 (0.34-0.94)	.03	0.78 (0.46-1.30)	.34
Noncardiovascular death					<.001				
	Normal	3737	472	8.1%		−		−	
	Underweight	303	60	15.1%		1.88 (1.44-2.46)	<.001	1.48 (1.12-1.94)	.005
	Overweight	1553	116	3.9%		0.54 (0.44-0.66)	<.001	0.64 (0.52-0.79)	<.001
	Obese	178	9	1.9%		0.37 (0.19-0.72)	.003	0.48 (0.25-0.94)	.03
Myocardial infarction					.20				
	Normal	3653	107	2.4%		−		−	
	Underweight	289	9	2.3%		1.25 (0.63-2.47)	.52	1.08 (0.54-2.15)	.83
	Overweight	1511	39	1.5%		0.81 (0.56-1.17)	.27	0.92 (0.63-1.33)	.65
	Obese	176	9	3.1%		1.68 (0.85-3.31)	.14	1.90 (0.96-3.77)	.07
Heart failure					.004				
	Normal	3725	397	7.7%		−		−	
	Underweight	301	32	9.3%		1.15 (0.80-1.65)	.44	0.91 (0.63-1.32)	.62
	Overweight	1551	129	6.1%		0.72 (0.59-0.87)	<.001	0.84 (0.69-1.03)	.10
	Obese	177	23	4.8%		1.16 (0.76-1.77)	.48	1.37 (0.90-2.09)	.15
Stroke					<.001				
	Normal	3679	340	6.9%		−		−	
	Underweight	299	35	9.9%		1.47 (1.04-2.08)	.03	1.28 (0.89-1.83)	.18
	Overweight	1525	112	4.4%		0.73 (0.59-0.91)	.005	0.79 (0.63-0.98)	.03
	Obese	177	11	4.8%		0.63 (0.35-1.15)	.13	0.69 (0.38-1.27)	.23
Repeat coronary revascularization					.44				
	Normal	3622	475	11.0%		−		−	
	Underweight	292	27	8.3%		0.79 (0.54-1.17)	.24	0.84 (0.56-1.24)	.37
	Overweight	1504	192	9.6%		0.91 (0.77-1.07)	.25	0.89 (0.75-1.06)	.19
	Obese	174	26	11.8%		1.08 (0.73-1.60)	.71	1.07 (0.72-1.59)	.74

Number of patients with event was counted throughout the entire follow-up period, whereas the cumulative incidence was estimated at 5 years. *CABG*, Coronary artery bypass grafting; *HR*, hazard ratio; *CI*, confidence interval.

**Figure 3:**
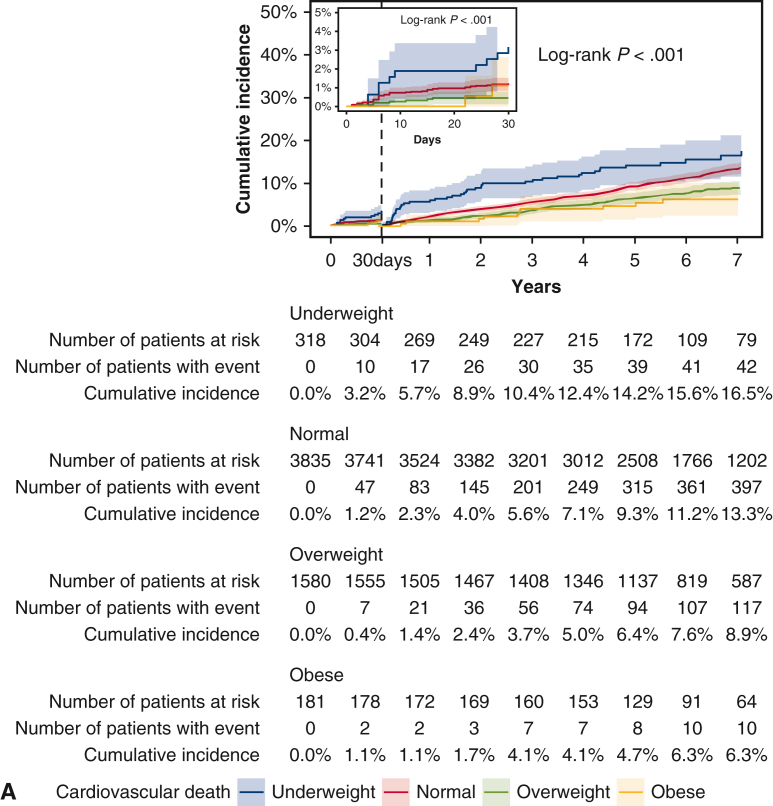

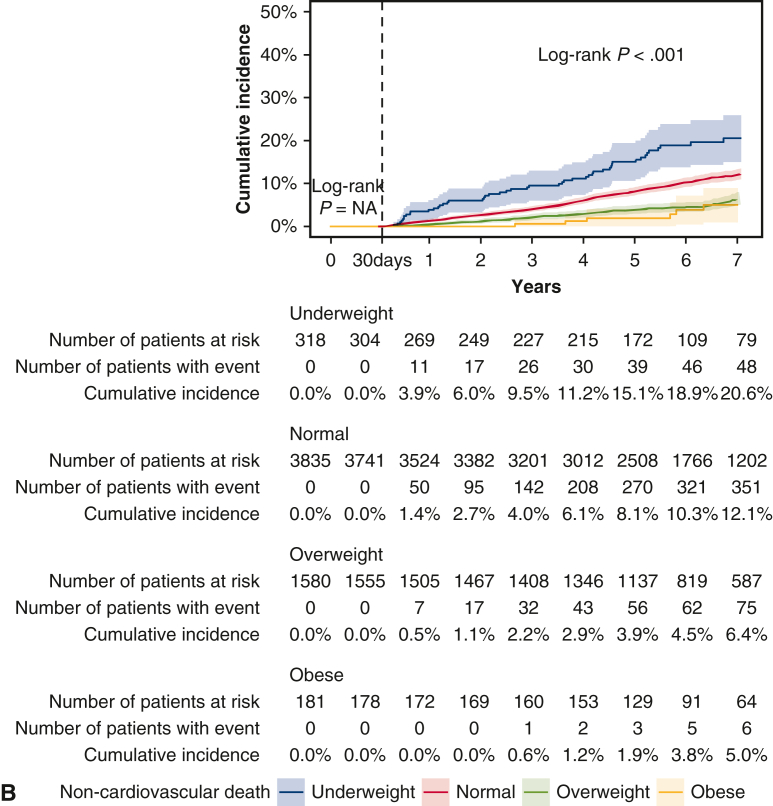
Kaplan-Meier event curves for clinical outcomes within and beyond 30 days after coronary artery bypass grafting. A, Cardiovascular death. B, Noncardiovascular death. 95% confidence intervals are shown via *shading*.

The cumulative 5-year incidence of cardiovascular death beyond 30 days after CABG was greater in the underweight group and lower in the overweight and obese groups than in the normal group (underweight: 14.2%, normal: 9.3%, overweight: 6.4%, and obesity: 4.7%; log-rank *P* < .001) (Table 3 and Figure 3, *A*). However, after adjusting for confounders, the risks of the underweight, overweight, and obese groups relative to the normal group were no longer significant for cardiovascular death (adjusted HR, 1.02; 95% CI, 0.77-1.37; *P* = .87, adjusted HR, 0.91; 95% CI, 0.76-1.08; *P* = .28, and adjusted HR, 0.78; 95% CI, 0.46-1.30; *P* = .34, respectively) (Table 3).

The cumulative 5-year incidence of noncardiovascular death beyond 30 days after CABG was greater in the underweight group and lower in the overweight and obese groups than in the normal group (underweight: 15.1%, normal: 8.1%, overweight: 3.9%, and obese: 1.9%; log-rank *P* < .001) (Table 3 and Figure 3, *B*). Even after adjusting for confounders, the greater risk of the underweight group relative to the normal group remained significant for noncardiovascular death (adjusted HR, 1.48; 95% CI, 1.12-1.94; *P* = .005), and the lower risk of the overweight and obese groups relative to the normal group remained significant for noncardiovascular death (adjusted HR, 0.64; 95% CI, 0.52-0.79; *P* < .001, and adjusted HR, 0.48; 95% CI, 0.25-0.94; *P* = .03) (Table 3).

**Figure E1:**
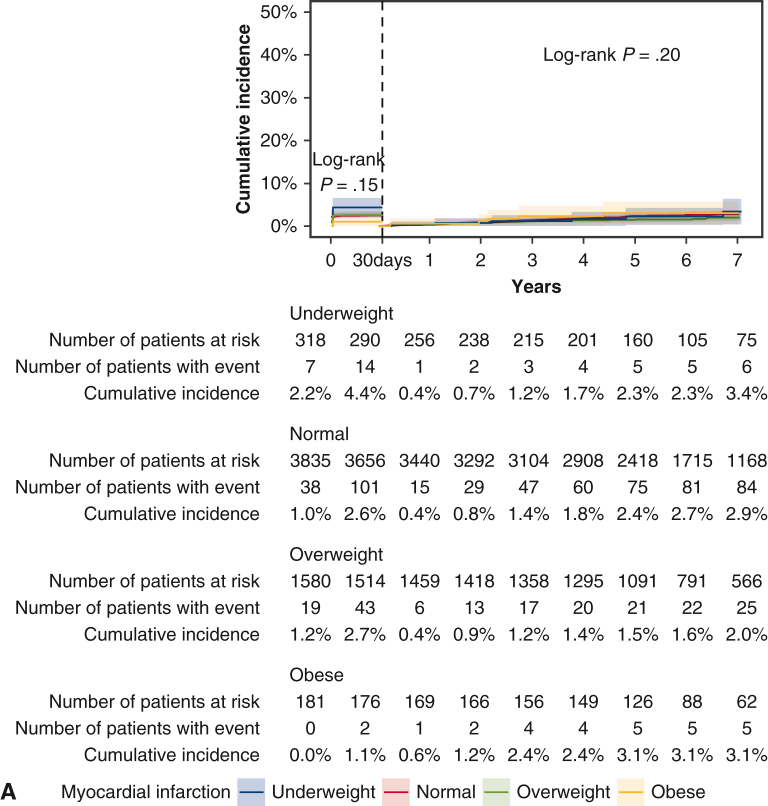

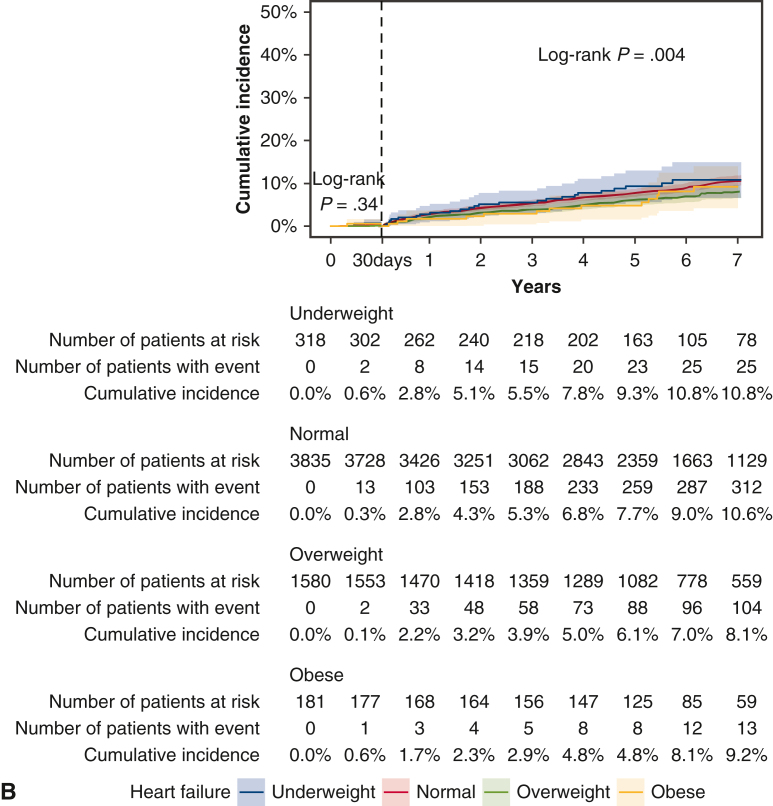

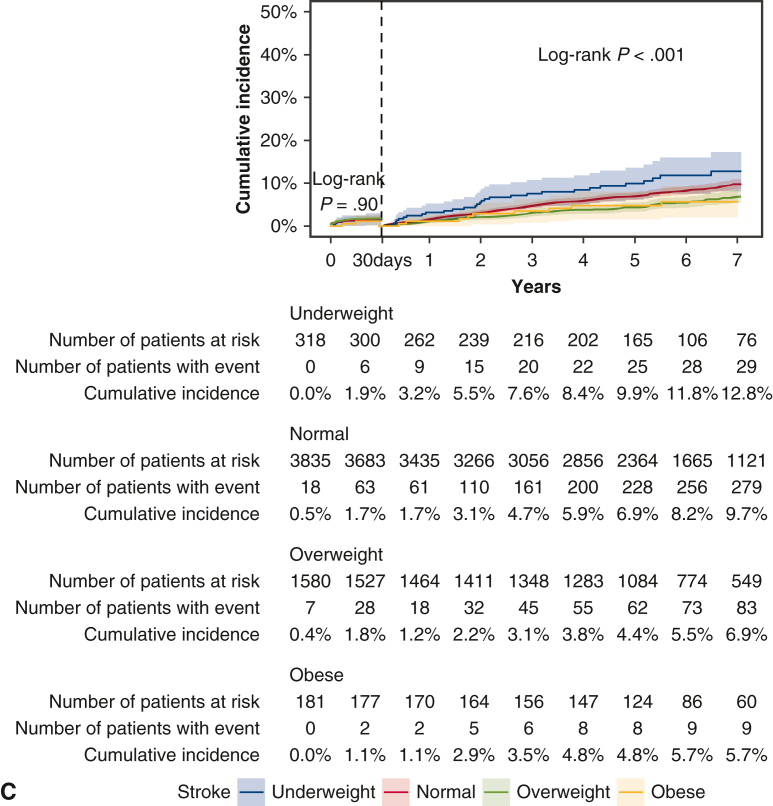

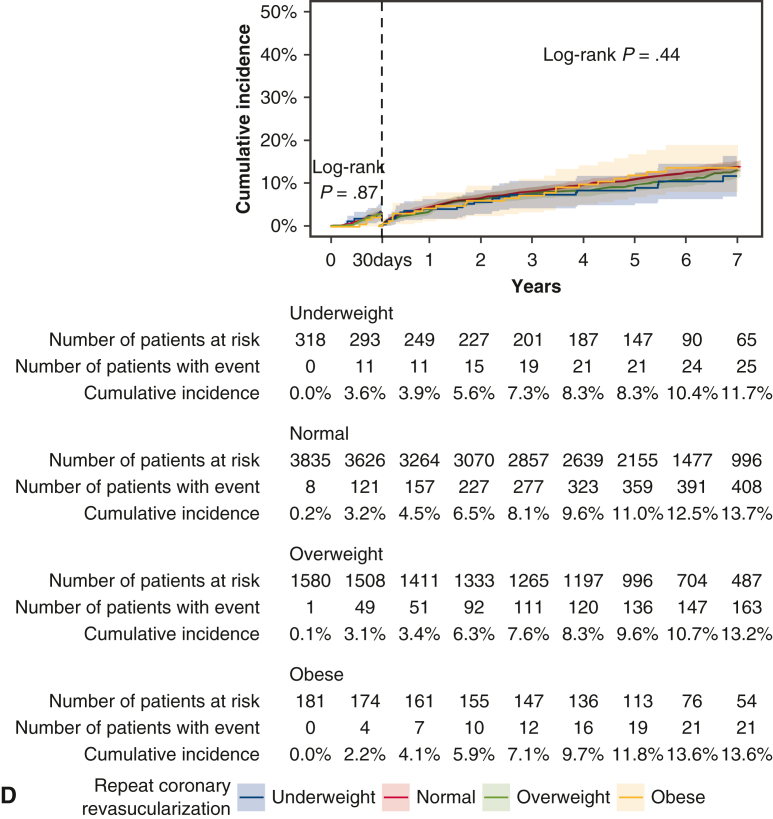
Kaplan-Meier event curves for clinical outcomes within and beyond 30 days after coronary artery bypass grafting. 95% CIs are shown via shading. A, Myocardial infarction. B, Heart failure. C, Stroke. D, Repeat coronary revascularization.

The cumulative 5-year incidences of heart failure and stroke, respectively, were greater in the underweight group than in the other groups (underweight: 9.3%, normal: 7.7%, overweight: 6.1%, and obese: 4.8%; log-rank *P* = .004, and underweight: 9.9%, normal: 6.9%, overweight: 4.4%, and obese: 4.8%; log-rank *P* < .001) (Table 3, Figure E1, *B* and *C*). The cumulative 5-year incidences of myocardial infarction and repeat revascularization did not differ across the four groups (Table 3, Figure E1, *A* and *D*). After adjusting for confounders, there was no excess risk in the underweight group relative to the normal group for heart failure, stroke, myocardial infarction, or repeat revascularization (Table 3).

### Analysis Stratified by Sex

**Figure E2:**
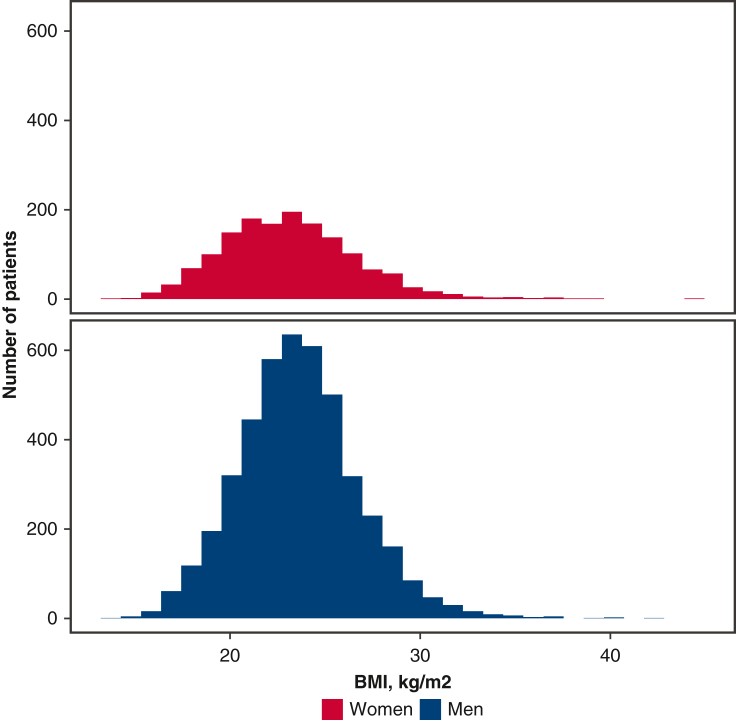
Distribution of BMI by sex. *BMI*, Body mass index.

There were several significant differences in baseline characteristics across the 4 groups between men and women (Table E1). The distribution of BMI was similar between men and women (Figure E2).

The cumulative 30-day incidence of all-cause death was greater in the underweight group in both men and women, and this trend was more prominent in men than in women (men: underweight: 4.0%, normal: 1.3%, overweight: 0.2%, and obese: 1.6%; log-rank *P* < .001; women: underweight: 1.7%, normal: 0.9%, overweight: 1.1%, and obese: 0.0%; log-rank *P* = .74) (Table E2). The cumulative 30-day incidence of myocardial infarction was significantly greater in the underweight group than in the other groups in men but not in women (men: underweight: 5.5%, normal: 2.6%, overweight: 2.5%, and obese: 0.8%; log-rank *P* = .04; women: underweight: 2.5%, normal: 2.7%, overweight: 3.3%, and obese: 1.9%; log-rank *P* = .90) (Table E2). The cumulative 30-day incidences of heart failure, stroke, and repeat coronary revascularization did not differ significantly between men and women across the 4 BMI groups.

**Figure 4:**
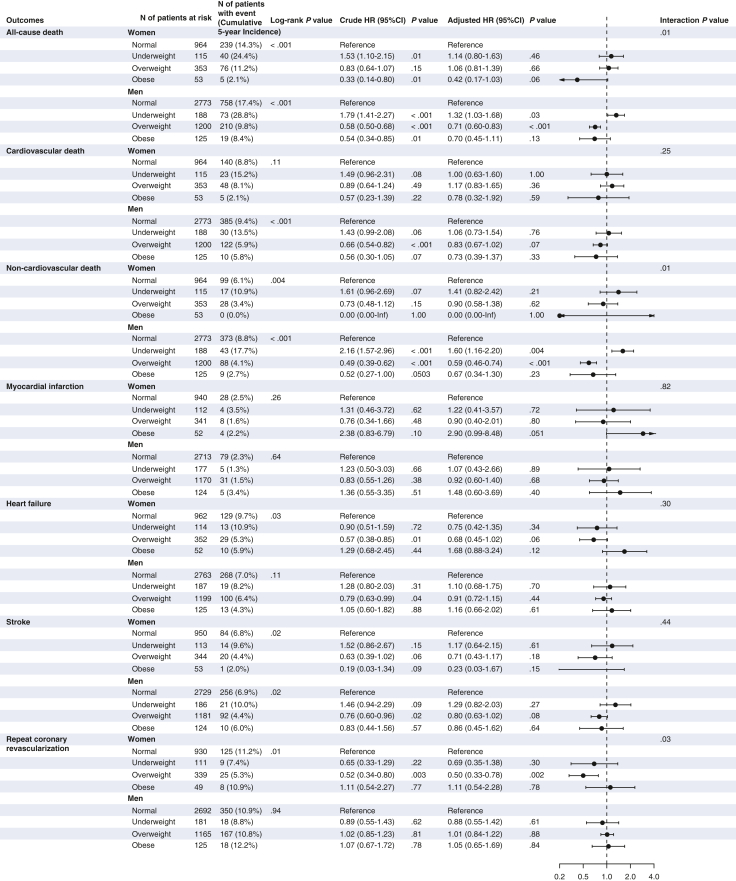
Subgroup analyses stratified by sex for clinical outcomes beyond 30 days after coronary artery bypass grafting. Number of patients with event was counted throughout the follow-up period, whereas the cumulative incidence was estimated at 5 years. *HR*, Hazard ratio; *CI*, confidence interval.

Regarding the long-term clinical outcomes beyond 30 days after CABG, the cumulative 5-year incidence of all-cause death was significantly greater in the underweight group than in the other groups in both men and women (men: underweight: 28.8%, normal: 17.4%, overweight: 9.8%, and obese: 8.4%; women: underweight: 24.4%, normal: 14.3%, overweight: 11.2%, and obese: 2.1%). However, after adjusting for confounders, there was a significantly greater risk for all-cause death in the underweight group relative to the normal group in men but not in women (men: adjusted HR, 1.32; 95% CI, 1.03-1.68; *P* = .03; women: adjusted HR, 1.14; 95% CI, 0.80-1.63; *P* = .46) (Figure 4). There was also a significantly lower risk for all-cause death in the overweight group relative to the normal group in men but not in women (men: adjusted HR, 0.71; 95% CI, 0.60-0.83; *P* < .001; women: adjusted HR, 1.06; 95% CI, 0.81-1.39; *P* < .001) (Figure 4). A significant interaction was found between sex and effect of BMI category on all-cause death (interaction *P* = .01).

The cumulative 5-year incidence of cardiovascular death was numerically greater in the underweight group than in the other groups in both men and women (men: underweight: 13.5%, normal: 9.4%, overweight: 5.9%, and obese: 5.8%, log-rank *P* < .001; women: underweight: 15.2%, normal: 8.8%, overweight: 8.1%, and obese: 2.1%, log-rank *P* = .11). However, after adjusting for confounders, the excess risk of cardiovascular death in the underweight group relative to the normal group was no longer significant in men or women (men: adjusted HR, 1.06; 95% CI, 0.73-1.54; *P* = .76; women: adjusted HR, 1.00; 95% CI, 0.63-1.60; *P* = 1.00; interaction *P* = .25) (Figure 4). The cumulative 5-year incidence of noncardiovascular death was significantly greater in the underweight group than in the other groups in both men and women (men: underweight: 17.7%, normal: 8.8%, overweight: 4.1%, and obese: 2.7%, log-rank *P* < .001; women: underweight: 10.9%, normal: 6.1%, overweight: 3.4%, and obese: 0.0%, log-rank *P* = .004). However, after adjusting for confounders, the greater risk of noncardiovascular death in the underweight group relative to the normal group was significant for men but not women (men: adjusted HR, 1.60; 95% CI, 1.16-2.20; *P* = .004; women: adjusted HR, 1.41; 95% CI, 0.82-2.42; *P* = .21, interaction *P* = .01) (Figure 4).

The adjusted risk of the underweight group relative to the normal group was not significant for myocardial infarction, heart failure, stroke, or repeat coronary revascularization in men or women (Figure 4).

## Discussion

The main findings of this study were as follows: (1) the cumulative 30-day incidence of all-cause death after CABG was greater in patients with underweight than in those without, especially in men; (2) the long-term risk for all-cause death beyond 30 days after CABG was greater in patients with underweight and lower in patients with overweight or with obesity compared with patients with normal BMI; and (3) the differences in long-term mortality risk depending on BMI were particularly prominent in men and mainly driven by the risk for noncardiovascular death.

Most previous studies evaluating the association between BMI and clinical outcomes after CABG either excluded patients with underweight or included only a very small number of patients with underweight. Our study included a much larger number of patients with underweight than previous studies and investigated its impact and sex differences.^4^^,^^10^

Regarding early postoperative outcomes, a previous single-center retrospective cohort study reported that patients with above-normal BMI who underwent CABG did not have increased in-hospital mortality (underweight: 8.7%, normal: 2.3%, overweight: 2.4%, class I obesity: 1.8%, class II/III obesity: 2.7%).^11^ In contrast, patients with underweight had a greater in-hospital mortality rate, although it was not statistically significant because of the small number of patients with underweight. Another single-center retrospective study from the Netherlands reported that among patients undergoing isolated CABG, having underweight is a predictor for early mortality (odds ratio, 2.63; 95% CI, 1.13-6.11).^4^ Consistent with these previous studies, the cumulative 30-day mortality rate after CABG in this study was significantly greater in patients with underweight than in those without, especially in men. Sarcopenia and malnutrition are reported to be associated with 30-day in-hospital major adverse cardiac and cerebral event rates after cardiac surgery.^12^ Although we speculated that nutritional status and sarcopenia may have an impact on our findings, our data lacked information regarding nutritional status or psoas muscle mass based on computed tomography. In addition, the underweight group may have included both individuals with cachexia and those with constitutional leanness, but it was difficult to clearly distinguish between these conditions on the basis of our data. If data on nutritional status were available, it might be possible to perform analyses to distinguish between cachexia and constitutional leanness. We would like to address these in future research.

According to several studies investigating the association between BMI and late outcomes after CABG, overweight and mild obesity did not increase late mortality, whereas morbid obesity (BMI ≥35) did increase late mortality.^3^^,^^4^^,^^13^ One of these reports, which included patients with underweight, reported a greater late mortality in patients with underweight, although this finding was not statistically significant (odds ratio, 1.38; 95% CI, 0.87-2.21).^4^ Consistent with these reports, in the current study, the long-term risk of all-cause death after CABG was significantly greater in patients with underweight and lower i in patients with overweight and with obesity. According to a pooled analysis of individual patient data from randomized controlled trials, obesity was associated with reduced graft failure at 1 year after CABG, which could be a factor related to greater long-term mortality risk in patients with underweight.^14^ However, in the present study, the greater long-term mortality risk in patients with underweight was mainly driven by noncardiovascular death, suggesting that the impact of BMI on long-term outcomes after CABG may be attributed mainly to sarcopenia and malnutrition. Underweight status—or its underlying causes, such as malnutrition—is thought to lead to decreased immune function and an increased risk of infection.^15^ Furthermore, we showed this trend was more prominent in men. Women have been reported to have worse outcomes than men after CABG.^16^ It has been reported that the increase in muscle mass related to an increase in body weight was greater in men than in women.^17^ Taken together, the smaller variance of muscle mass in women may explain the different impact of BMI on outcomes between men and women after CABG. We speculated that male patients with underweight may have greater incidences of sarcopenia and malnutrition and experience noncardiovascular death such as from infection. Regarding generalizability, our cohort was exclusively Japanese, and the BMI distribution in the Japanese population is reported to differ from that of Western populations.^18^ However, the biological mechanisms by which being underweight affects post-CABG outcomes are considered universal. Preoperative nutritional optimization and enhanced post-CABG surveillance might improve clinical outcomes in patients with underweight after CABG.

This study has several limitations. First, because of the observational nature of the study, there might be unmeasured confounders. Our data lacked information on frailty, nutritional status, and socioeconomic factors, and these unmeasured confounders may have influenced the results. However, we believe that the confounding factors we adjusted for—anemia, malignancy, and end-stage renal disease—are strongly associated with nutritional status and frailty, and therefore we reduced the impact of unmeasured confounders to some extent. Second, the number of patients in the obese group was smaller than the numbers in the other BMI categories, and the adjusted HR in this group showed a wide 95% CI. Also, our study included a very small number of patients with BMI ≥35. According to a previous study, morbid obesity is reported to be a predictor for late mortality.^4^ However, we could not evaluate the impact of morbid obesity. Third, because the study population included 3 cohorts from different time periods, there may be differences in patient backgrounds and surgical outcomes. However, the surgical procedure of CABG itself has not changed substantially, and we adjusted for as many confounders as possible. We conducted a stratified analysis by cohort, and the interaction between BMI and cohort was not significant. Therefore, we considered the impact of time period to be limited. Fourth, despite having a greater proportion of patients with low ejection fraction and previous myocardial infarction, the underweight group had less use of aspirin, statins, beta-blockers, and angiotensin-converting enzyme inhibitors or angiotensin II receptor blockers, which might have had an impact on the results. Fifth, in the stratified analysis by sex, the underweight group in women may be underpowered. Sixth, our results cannot be applied to urgent/emergent CABG populations because we excluded patients with acute myocardial infarction. Finally, there was no prespecified plan to adjust for multiple comparisons. Therefore, 95% CIs were not adjusted for multiple testing. In conclusion, being underweight was associated with increased short- and long-term mortality after CABG, especially in men, whereas being overweight or obese was associated with decreased long-term mortality after CABG.

### Webcast

You can watch a Webcast of this AATS meeting presentation by going to: https://www.aats.org/resources/is-being-underweight-with-a-bm-9684.

**Figure:**
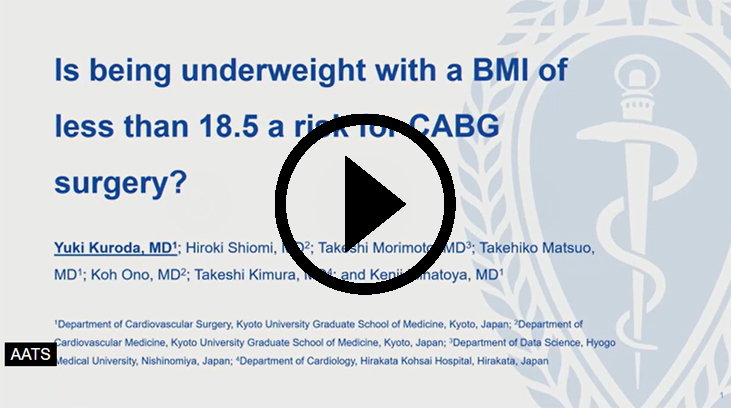

### Audio

You can listen to the discussion audio of this article by going to the Supplemental Materials.

## Conflict of Interest Statement

The authors reported no conflicts of interest.

The *Journal* policy requires editors and reviewers to disclose conflicts of interest and to decline handling or reviewing manuscripts for which they may have a conflict of interest. The editors and reviewers of this article have no conflicts of interest.

## Figures and Tables

**Table E1:** Baseline characteristics and management stratified by sex

Characteristic	Women, N = 1516					Men, N = 4398				
	Underweight n = 118	Normal n = 985	Overweight n = 360	Obese n = 53	*P* value	Underweight n = 200	Normal n = 2850	Overweight n = 1220	Obese n = 128	*P* value
Clinical characteristics										
Body mass index, kg/m^2^	17.4 (0.9)	22.0 (1.7)	26.8 (1.3)	32.6 (2.8)	<.001	17.5 (0.9)	22.4 (1.7)	26.7 (1.3)	32.1 (2.3)	<.001
Age, y	71.4 (10.2)	70.7 (8.4)	69.9 (8.5)	66.5 (10.6)	.02	70.4 (8.9)	68.2 (9.1)	65.5 (9.5)	59.8 (12.4)	<.001
Age ≥75 y	52 (44%)	359 (36%)	110 (31%)	16 (30%)	.03	73 (37%)	730 (26%)	220 (18%)	16 (13%)	<.001
Unstable angina	12 (10%)	72 (7.3%)	16 (4.4%)	1 (1.9%)	.051	9 (4.5%)	146 (5.1%)	49 (4.0%)	2 (1.6%)	.15
Hypertension	79 (67%)	791 (80%)	320 (89%)	48 (91%)	<.001	140 (70%)	2149 (75%)	985 (81%)	111 (87%)	<.001
Diabetes	56 (47%)	526 (53%)	198 (55%)	28 (53%)	.56	85 (43%)	1346 (47%)	580 (48%)	65 (51%)	.48
On insulin therapy	25 (21%)	207 (21%)	72 (20%)	9 (17%)	.89	31 (16%)	426 (15%)	135 (11%)	22 (17%)	.01
Current smoker	13 (11%)	96 (9.8%)	43 (12%)	6 (11%)	.71	64 (32%)	747 (27%)	333 (28%)	37 (29%)	.33
Heart failure	29 (25%)	159 (16%)	61 (17%)	6 (11%)	.09	37 (19%)	362 (13%)	145 (12%)	12 (9.4%)	.045
LVEF, %	55.8 (14.9)	61.3 (14.6)	63.2 (12.7)	66.3 (11.5)	<.001	54.5 (15.7)	58.1 (14.4)	60.4 (13.0)	58.1 (13.2)	<.001
≤40%	21 (18%)	95 (9.6%)	18 (5.0%)	2 (3.8%)	<.001	37 (19%)	357 (13%)	102 (8.4%)	15 (12%)	<.001
Severe MR	6 (5.5%)	39 (4.3%)	11 (3.3%)	2 (3.9%)	.74	10 (5.6%)	82 (3.1%)	25 (2.2%)	1 (0.8%)	.03
Previous myocardial infarction	43 (36%)	222 (23%)	65 (18%)	8 (15%)	<.001	61 (31%)	848 (30%)	323 (26%)	36 (28%)	.19
Previous stroke	18 (15%)	162 (16%)	56 (16%)	3 (5.7%)	.22	49 (25%)	527 (18%)	213 (17%)	23 (18%)	.13
Peripheral vascular disease	17 (14%)	129 (13%)	34 (9.5%)	6 (11%)	.29	50 (25%)	530 (19%)	137 (11%)	20 (16%)	<.001
Atrial fibrillation	11 (9.3%)	78 (7.9%)	25 (6.9%)	6 (11%)	.65	27 (14%)	318 (11%)	121 (9.9%)	15 (12%)	.41
eGFR, mL/min/1.73 m^2^	46.2 (23.5)	55.6 (27.5)	62.8 (25.4)	78.5 (38.0)	<.001	48.3 (26.1)	59.7 (25.4)	67.8 (27.5)	75.8 (38.3)	<.001
eGFR <30, without hemodialysis	15 (13%)	97 (9.8%)	26 (7.2%)	2 (3.8%)	.12	12 (6.0%)	152 (5.3%)	50 (4.1%)	7 (5.5%)	.36
Hemodialysis	12 (10%)	64 (6.5%)	12 (3.3%)	0 (0%)	.01	35 (18%)	197 (6.9%)	41 (3.4%)	4 (3.1%)	<.001
Anemia (hemoglobin <11.0 g/dL)	57 (48%)	351 (36%)	82 (23%)	10 (19%)	<.001	58 (29%)	431 (15%)	86 (7.0%)	8 (6.3%)	<.001
Thrombocytopenia (platelet <100 × 10^9^/L)	1 (0.8%)	13 (1.3%)	4 (1.1%)	0 (0%)	.82	3 (1.5%)	43 (1.5%)	13 (1.1%)	3 (2.3%)	.56
Chronic obstructive pulmonary disease	3 (2.5%)	25 (2.5%)	9 (2.5%)	2 (3.8%)	.96	13 (6.5%)	108 (3.8%)	23 (1.9%)	1 (0.8%)	<.001
Liver cirrhosis	2 (1.7%)	36 (3.7%)	7 (1.9%)	1 (1.9%)	.30	12 (6.0%)	95 (3.3%)	27 (2.2%)	1 (0.8%)	.01
Malignancy	11 (9.3%)	70 (7.1%)	33 (9.2%)	5 (9.4%)	.54	27 (14%)	288 (10%)	94 (7.7%)	8 (6.3%)	.01
Procedural characteristics										
Extent of coronary artery disease					.85					.59
One-vessel disease	2 (1.7%)	14 (1.4%)	7 (1.9%)	1 (1.9%)		3 (1.5%)	65 (2.3%)	19 (1.6%)	3 (2.3%)	
Two-vessel disease	19 (16%)	122 (12%)	47 (13%)	8 (15%)		27 (14%)	357 (13%)	165 (14%)	16 (13%)	
Three-vessel disease	63 (53%)	544 (55%)	212 (59%)	28 (53%)		103 (52%)	1497 (53%)	671 (55%)	73 (57%)	
Left main coronary artery disease	34 (29%)	305 (31%)	94 (26%)	16 (30%)		67 (34%)	931 (33%)	365 (30%)	36 (28%)	
Cardiopulmonary bypass use	59 (50%)	435 (44%)	154 (43%)	28 (53%)	.33	93 (47%)	1284 (45%)	558 (46%)	60 (47%)	.94
Internal thoracic artery use	109 (92%)	932 (95%)	346 (96%)	49 (92%)	.35	186 (93%)	2749 (96%)	1186 (97%)	123 (96%)	.03
Medications at discharge										
Aspirin	104 (89%)	897 (91%)	329 (92%)	51 (96%)	.47	178 (89%)	2618 (92%)	1128 (93%)	125 (98%)	.03
Thienopyridine	17 (15%)	125 (13%)	47 (13%)	7 (13%)	.96	19 (9.5%)	404 (14%)	155 (13%)	11 (8.6%)	.07
Cilostazol	8 (6.8%)	88 (9.0%)	25 (7.0%)	3 (5.7%)	.53	13 (6.5%)	255 (9.0%)	86 (7.1%)	4 (3.1%)	.02
Statins	29 (25%)	360 (37%)	161 (45%)	32 (60%)	<.001	39 (20%)	873 (31%)	456 (37%)	68 (53%)	<.001
ACE-I/ARB	25 (21%)	280 (28%)	109 (30%)	26 (49%)	.003	51 (26%)	691 (24%)	338 (28%)	39 (30%)	.07
Beta-blockers	22 (19%)	241 (25%)	88 (25%)	20 (38%)	.07	42 (21%)	709 (25%)	362 (30%)	37 (29%)	.004
Nitrates	45 (38%)	350 (36%)	138 (38%)	17 (32%)	.67	74 (37%)	981 (34%)	388 (32%)	40 (31%)	.27
Calcium-channel blockers	49 (42%)	536 (55%)	219 (61%)	29 (55%)	.004	89 (45%)	1462 (51%)	610 (50%)	66 (52%)	.28
Oral anticoagulants	40 (34%)	355 (36%)	124 (35%)	18 (34%)	.93	75 (38%)	1100 (39%)	439 (36%)	50 (39%)	.47
Warfarin	40 (34%)	354 (36%)	124 (35%)	18 (34%)	.94	75 (38%)	1093 (38%)	436 (36%)	50 (39%)	.47
DOAC	0 (0%)	1 (0.1%)	0 (0%)	0 (0%)	.91	0 (0%)	7 (0.2%)	3 (0.2%)	0 (0%)	.85

Continuous variables are expressed as mean (standard deviation). Categorical variables are expressed as number (percentage). *LVEF,* Left ventricular ejection fraction; *MR*, mitral regurgitation; *eGFR*, estimated glomerular filtration rate; *ACE-I*, angiotensin-converting enzyme inhibitor; *ARB*, angiotensin II receptor blocker; *DOAC*, direct oral anticoagulant.

**Table E2:** Clinical outcomes within 30 days after CABG stratified by sex

Outcome	Subgroup/group	n	No. patients with event	Cumulative 30-d incidence	Log-rank *P* value
All-cause death	Women				.74
	Normal	985	9	0.9%	
	Underweight	118	2	1.7%	
	Overweight	360	4	1.1%	
	obese	53	0	0.0%	
	Men				<.001
	Normal	2850	38	1.3%	
	Underweight	200	8	4.0%	
	Overweight	1220	3	0.2%	
	Obese	128	2	1.6%	
Cardiovascular death	Women				
	Normal	985	9	0.9%	.74
	Underweight	118	2	1.7%	
	Overweight	360	4	1.1%	
	Obese	53	0	0.0%	
	Men				
	Normal	2850	38	1.3%	<.001
	Underweight	200	8	4.0%	
	Overweight	1220	3	0.2%	
	Obese	128	2	1.6%	
Myocardial infarction	Women				.90
	Normal	985	27	2.7%	
	Underweight	118	3	2.5%	
	Overweight	360	12	3.3%	
	Obese	53	1	1.9%	
	Men				.04
	Normal	2850	74	2.6%	
	Underweight	200	11	5.5%	
	Overweight	1220	31	2.5%	
	Obese	128	1	0.8%	
Heart failure	Women				.15
	Normal	985	2	0.2%	
	Underweight	118	1	0.9%	
	Overweight	360	1	0.3%	
	Obese	53	1	1.9%	
	Men				.33
	Normal	2850	11	0.4%	
	Underweight	200	1	0.5%	
	Overweight	1220	1	0.1%	
	Obese	128	0	0.0%	
Stroke	Women				.42
	Normal	985	14	1.4%	
	Underweight	118	2	1.7%	
	Overweight	360	9	2.5%	
	Obese	53	0	0.0%	
	Men				.96
	Normal	2850	49	1.7%	
	Underweight	200	4	2.0%	
	Overweight	1220	19	1.6%	
	Obese	128	2	1.6%	
Repeat coronary revascularization	Women				.50
	Normal	985	34	3.5%	
	Underweight	118	4	3.4%	
	Overweight	360	14	3.9%	
	Obese	53	4	7.5%	
	Men				.23
	Normal	2850	87	3.1%	
	Underweight	200	7	3.6%	
	Overweight	1220	35	2.9%	
	Obese	128	0	0.0%	

There was no patient with noncardiovascular death within 30 days after CABG. *CABG*, Coronary artery bypass grafting.

## Appendix E2 List of Clinical Research Coordinators

### Research Institute for Production Development

S. Arimura, Y. Fujino, M. Hanazawa, C. Hibi, R. Kato, Y. Kinoshita, K. Kitagawa, M. Kitamura, T. Kuwahara, S. Maeda, I. Miki, S. Minematsu, S. Nishida, N. Okamoto, A. Saeki, Y. Sato, H. Sasae, A. Takahashi, E. Takinami, S. Tezuka, M. Tsuda, M. Tsumori, Y. Uchida, M. Yamauchi, Y. Yamamoto, I. Yamazaki, M. Yoshimoto.

## Appendix E3 Definitions of Baseline Characteristics and Outcome Measures

Baseline characteristics, such as hypertension, current smoking, heart failure, previous myocardial infarction, and chronic obstructive pulmonary disease were regarded as present when these diagnoses were documented in the hospital charts. Diabetes was defined as treatment with oral hypoglycemic agents or insulin, previous clinical diagnosis of diabetes, glycated hemoglobin level ≥6.5%, or nonfasting blood glucose level ≥200 mg/dL. Peripheral vascular disease was regarded as present when carotid, aortic, or other peripheral vascular diseases were being treated or scheduled for surgical or endovascular interventions. Renal function was evaluated by the estimated glomerular filtration rate calculated by the Modification of Diet in Renal Disease formula modified for Japanese patients.^E1^

Death was regarded as cardiovascular in origin unless obvious noncardiovascular causes could be identified. Any death during the index hospitalization for coronary artery bypass grafting was regarded as cardiovascular death. Myocardial infarction was defined according to the Academic Research Consortium definition.^E2^ Heart failure was defined as de novo hospitalization or prolongation of hospitalization attributable to heart failure requiring intravenous treatment. Repeat coronary revascularization included either percutaneous coronary intervention or coronary artery bypass grafting for any reasons. The clinical event committee adjudicated all the events for the outcome measures.

## Supplementary Data

Audio 1 This is an audio recording of the presentation of the associated abstract and discussion. The abstract presentation ends at 7:10 and the discussion begins at 7:15.

## Appendix E1 List of Participating Centers and Investigators

### CREDO-Kyoto Registry Cohort-1

Kyoto University Hospital: T. Kimura, H. Shiomi.

Kishiwada City Hospital: M. Matsuda, Y. Takeuchi, H. Mitsuoka.

Tenri Hospital: Y. Nakagawa, T. Tamura, T. Konishi, S. Ootani.

Hyogo Prefectural Amagasaki Hospital: Y. Takatsu, R. Taniguchi, K. Kataoka.

Kitano Hospital: M. Inoko, R. Nohara, K. Nakano, S. Miyamoto, N. Kunihiko.

Koto Memorial Hospital: T. Murakami, T. Takeda, K. Ishida.

Kokura Memorial Hospital: M. Nobuyoshi, K. Yamaji, H. Yasumoto.

Maizuru Kyosai Hospital: M. Kato, R. Tatami.

Nara Hospital, Kinki University Faculty of Medicine: M. Shirotani, R. Hattori.

Kobe City Medical Center General Hospital: Y. Furukawa, N. Ehara, Y. Kihara.

Nishi Kobe Medical Center: H. Eizawa, H. Kato.

Kansai Denryoku Hospital: K. Ishii, T. Aoyama, T. Sakurai, M. Kawanami, T. Suyama.

Osaka Red Cross Hospital: M. Tanaka, T. Inada.

University of Fukui Hospital: H. Uzui, A. Nakano.

Shizuoka City Shizuoka Hospital: T. Onodera, A. Takizawa.

Hamamatsu Rosai Hospital: E. Shinoda, M. Takahashi.

Shiga University of Medical Science Hospital: M. Horie, T. Yamamoto, H. Takashima.

Japanese Red Cross Society Wakayama Medical Center: T. Tamura, M. Toyofuku, H. Kotoura, A. Miura.

Shimabara Hospital: M. Takahashi, Y. Matoba.

Kagoshima University Medical and Dental Hospital: T. Takumi, C. Tei, S. Hamasaki.

Shizuoka General Hospital: O. Doi, H. Kambara, S. Kaburagi.

Kurashiki Central Hospital: K. Mitsudo, K. Kadota.

Mitsubishi Kyoto Hospital: S. Miki, T. Mizoguchi, Y. Akira.

Kumamoto University Hospital: H. Ogawa, K. Sugamura, S. Sugiyama.

Shimada Municipal Hospital: T. Aoyama, K. Doyama, M. Araki.

Juntendo University Shizuoka Hospital: S. Suwa.

### CREDO-Kyoto Registry Cohort-2

Kyoto University Hospital: T. Kimura, H. Shiomi, R. Sakata, T. Ikeda, A. Marui.

Kishiwada City Hospital: M. Matsuda, H. Mitsuoka, M. Onoe.

Tenri Hospital: Y. Nakagawa, K. Yamanaka.

Hyogo Prefectural Amagasaki Hospital: H. Fujiwara, Y. Takatsu, R. Taniguchi, K. Fujiwara, N. Ohno.

Kitano Hospital: R. Nohara.

Koto Memorial Hospital: T. Murakami, T. Takeda.

Kokura Memorial Hospital: M. Nobuyoshi, M. Iwabuchi, M. Hanyu.

Maizuru Kyosai Hospital: R. Tatami, T. Matsushita.

Nara Hospital, Kinki University Faculty of Medicine: M. Shirotani, N. Nishiwaki, Y. Yoshida.

Kobe City Medical Center General Hospital: T. Kita, Y. Furukawa, N. Ehara, Y. Okada, M. Nasu.

Nishi-Kobe Medical Center: H. Kato, H. Eizawa.

Kansai Denryoku Hospital: K. Ishii.

Osaka Red Cross Hospital: M. Tanaka, S. Nakayama.

University of Fukui Hospital: J. D. Lee, A. Nakano, K. Tanaka, T. Koshiji, K. Morioka.

Shizuoka City Shizuoka Hospital: A. Takizawa, M. Shimamoto, F. Yamazaki.

Hamamatsu Rosai Hospital: M. Takahashi, J. Nishizawa.

Shiga University of Medical Science Hospital: M. Horie, H. Takashima.

Japanese Red Cross Wakayama Medical Center: T. Tamura, M. Aota.

Shimabara Hospital: M. Takahashi, T. Tabata.

Kagoshima University Medica and Dental Hospital: C. Tei, S. Hamasaki, Y. Imoto, H. Yamamoto.

Shizuoka General Hospital: H. Kambara, O. Doi, S. Kaburagi, K. Matsuda, M. Nara.

Kurashiki Central Hospital: K. Mitsudo, K. Kadota, T. Komiya.

Mitsubishi Kyoto Hospital: S. Miki, T. Mizoguchi, H. Nakajima.

Kumamoto University Hospital: H. Ogawa, S. Sugiyama, M. Kawasuji, S. Moriyama.

Shimada Municipal Hospital: R. Hattori, T. Aoyama, M. Araki.

Juntendo University Shizuoka Hospital: S. Suwa, K. Tanbara.

### CREDO-Kyoto Registry Cohort-3

Kyoto University Hospital: T. Kimura, H. Shiomi, K. Minatoya, K. Yamazaki.

Kishiwada City Hospital: M. Matsuda, T. Uegaito, T. Ogawa.

Tenri Hospital: T. Tamura, A. Iwakura.

Hyogo Prefectural Amagasaki General Medical Center: Y. Sato, R. Taniguchi, N. Ohno.

Kitano Hospital: M. Inoko, M. Hanyu.

Koto Memorial Hospital: Tomoyuki Murakami, T. Takeda.

Kokura Memorial Hospital: K. Ando, T. Domei, K. Soga, A. Marui.

Kindai University Nara Hospital: M. Shirotani, N. Tamura.

Kobe City Medical Center General Hospital: Y. Furukawa, N. Ehara, T. Koyama.

Kobe City Nishi-Kobe Medical Center: H. Eizawa.

Kansai Denryoku Hospital: K. Ishii, E. Tada.

Osaka Red Cross Hospital: M. Tanaka, T. Inada, S. Nakayama.

Shizuoka City Shizuoka Hospital: T. Onodera, R. Nawada, F. Yamazaki, Y. Terai.

Hamamatsu Rosai Hospital: E. Shioda, M. Yamada, J. Nishizawa.

Shiga University of Medical Science Hospital: T. Yamamoto, H. Sakai.

Japanese Red Cross Wakayama Medical Center: T. Tamura, M. Toyofuku, N. Kanemitsu, H. Hara.

Shimabara Hospital: M. Takahashi.

Shizuoka General Hospital: H. Sakamoto, T. Tada, H. Tsuneyoshi.

Kurashiki Central Hospital: K. Kadota, T. Tada, T. Komiya.

Mitsubishi Kyoto Hospital: S. Miki, K. Kaneda, J. Esaki.

Shimada Municipal Hospital: T. Aoyama.

Juntendo University Shizuoka Hospital: S. Suwa, K. Tanbara.
